# The Proper Diagnosis of Thrombophilic Status in Preventing Fetal Growth Restriction

**DOI:** 10.3390/diagnostics13030512

**Published:** 2023-01-31

**Authors:** Bianca-Margareta Mihai, Teodor Salmen, Ana-Maria Cioca, Roxana-Elena Bohîlțea

**Affiliations:** 1 Doctoral School, “Carol Davila” University of Medicine and Pharmacy, 37 Dionisie Lupu, 020021 Bucharest, Romania; 2 Department of Obstetrics and Gynecology, Filantropia Hospital, 11-13 Ion Mihalache Blv., Sector 1, 011171 Bucharest, Romania; 3 Department of Obstetrics and Gynecology, “Carol Davila” University of Medicine and Pharmacy Bucharest, 37 Dionisie Lupu, 020021 Bucharest, Romania

**Keywords:** fetal growth restriction, thrombophilia, factor V Leiden, MTHFR C677T mutation, protein S deficiency, antithrombin deficiency, factor VII polymorphism

## Abstract

Fetal growth restriction is an important part of monitoring a pregnancy. Because guidelines or diagnostic criteria for either minor or major thrombophilia are scarce, this systematic review aims to summarize the present knowledge in the field. We performed the CRD42022376006 protocol in Prospero with a systematic literature search in PubMed and Web of Science databases and included original full-text articles (randomized control trials and clinical trials) from the last 10 years, published in English, and with the “thrombophilia AND (pregnancy OR diagnostic criteria) AND fetal growth restriction” criteria. After two researchers extracted the articles of interest, they were assessed using the Newcastle–Ottawa Scale and eight articles were included. The elements from the thrombophilia diagnostic predict IUGR, factor V Leiden mutation, MTHFR C667T mutation, protein S deficiency, antithrombin deficiency, factor VII polymorphism, and antiphospholipid antibodies, while the association of protein C, PAI-1 and certain combinations of mutations are still under debate and require the collection of more data. The present systematic review provides an extensive picture of the actual knowledge about thrombophilia diagnosis and its links with pregnancy complications, such as intrauterine growth restriction, despite its limitation in the inclusion of other actually debated disorders such as PAI-1 mutation, protein C deficiency and other thrombophilia types.

## 1. Introduction

Fetal growth restriction (FGR) is a term describing a fetus that is unable to achieve its full genetic growth potential in intrauterine life due to fetal, maternal and/or placental causes [1]. Fetal factors implied in the origin of FGR are represented by: genetic syndromes (13, 18, 21 trisomies), chromosomal anomalies, intrauterine infections (cytomegalovirus, rubella virus, varicella zoster virus infection, toxoplasma infection), inborn errors of metabolism, or multiple gestation pregnancies, especially in monochorionic twins, complicated by twin-to-twin transfusion syndrome [1]. Potential placental disorders responsible for FGR include placental insufficiency, infarction or abruptio placentae, placental hemangioma or chorioangioma, the circumvallate shape of the placenta; also, umbilical cord anomalies have been implicated in some studies as being associated with FGR (marginal or velamentous cord insertion, single umbilical artery) [2,3,4,5]. Maternal factors include hypertension, insulin-dependent diabetes mellitus complicated with vasculopathy, renal disorders, autoimmune diseases (antiphospholipid antibody syndrome, collagenoses), hyperhomocysteinemia, severe anaemia, acquiring hereditary thrombophilia, chronic malnutrition, drug consumption (tobacco, heroin, cocaine, alcohol), use of teratogenic medication, depression and elevated stress levels [1,6,7].

FGR complicates up to 10% of pregnancies, representing a primary cause for morbidity and mortality in infancy [8]. Postpartum, newborns with intrauterine growth restriction are at increased risk of hypoglycemia, hypothermia, hyperbilirubinemia, necrotizing enterocolitis, intraventricular hemorrhage, seizures, respiratory distress syndrome, sepsis and neonatal death in extreme cases [9]. The predisposition of certain diseases also remains substantial in adulthood, including cognitive or neurological impairment as well as cardiovascular or endocrine disorders such as stroke, coronary artery disease, dyslipidemia or even diabetes mellitus [2,10].

FGR is diagnosed using ultrasonographic measurements, or estimated fetal weight (EFW) when the EFW is below the 10th percentile for the gestational age. Further measurements should be performed (amniotic fluid index and umbilical artery Doppler velocimetry), as well as a detailed ultrasonographic evaluation of the fetus in order to exclude structural anomalies or genetic syndromes [2]. The Delphi consensus [11] classified FGR at under 32 gestational weeks as early, and FGR occurring after 32 weeks as late FGR. The criteria for early FGR are represented by: abdominal circumference (AC)/EFW < 3rd centile or umbilical artery absent and diastolic flow or AC/EFW < 10th centile combined with uterine artery pulsatility index (UtA-PI) > 95th centile and/or umbilical artery pulsatility index (UA-PI) > 95th centile. Late FGR is defined as AC/EFW < 3rd centile or at least two criteria out of the following three: AC/EFW crossing centiles > 2 quartiles on growth centiles, AC/EFW < 10th centile and cerebroplacental ratio < 5th centile or UA-PI > 95th centile.

At present, there is a heterogenous opinion between the society guidelines regarding the diagnosis of high-risk and low-risk types of thrombophilia in pregnancy, although there is a lack of consensus regarding further management of these pregnancies. American College of Obstetricians and Gynecologists (ACOG) [12] recommends screening for factor V Leiden and prothrombin G20210A mutations and protein C, protein S and antithrombin deficiencies for women with a personal history of venous thromboembolism (VTE), while for women with recurrent pregnancy loss or stillbirth they recommend testing the antiphospholipid antibodies. The Society of Obstetric Medicine of Australia and New Zealand [13] has classified inherited thrombophilia types as significant (antithrombin deficiency, factor V Leiden homozygous mutation, combined heterozygous mutation of factor V Leiden and prothrombin, protein S deficiency, protein C deficiency) and weak (factor V Leiden heterozygous mutation, prothrombin heterozygous mutation). The Society of Obstetricians and Gynecologist of Canada does not recommend routine screening for inherited thrombophilia in women with a first episode of VTE which occurred and was diagnosed in pregnancy while indicating protein C, S and antithrombin deficiencies after a VTE during pregnancy if a family history of protein C, S or antithrombin deficiency is present or if the thrombus is located in an uncommon site [14]. In Europe, for the moment, there is no guideline regarding thrombophilia in pregnancy.

Despite these discrepancies, the aim of our study was to determine whether other types of inherited or acquired thrombophilia influence the fetal outcome, especially if fetal growth restriction occurs in the presence of often ignored thrombophilia types by highlighting the fetal and neonatal outcomes.

## 2. Materials and Methods

**Figure 1:**
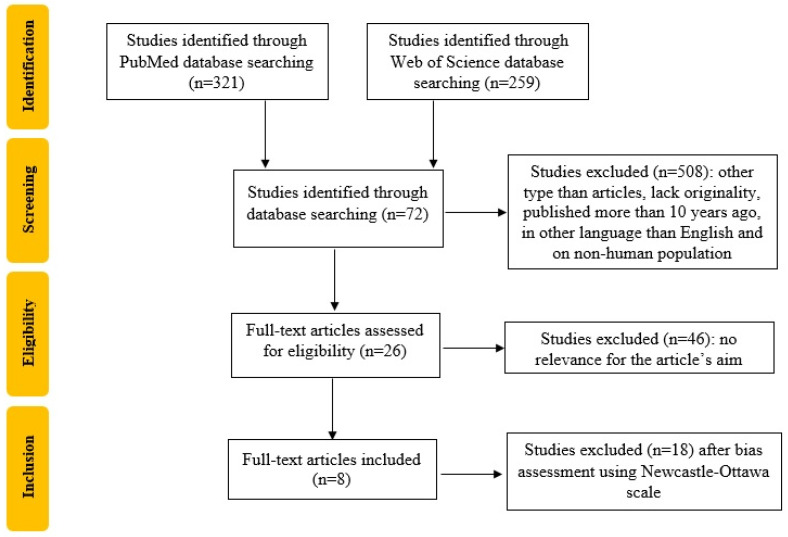
Study selection process flowchart according to the PRISMA guidelines.

We registered a systematic review protocol under the number CRD42022376006 in Prospero that followed the recommendations of Preferred Reporting Items for Systematic Reviews and Meta-Analyses (PRISMA). Furthermore, we used the Population, Intervention, Comparison, Outcome, and Study Design (PICOS) strategy to guide our study rationale and to conduct a clear, useful and systematic literature search. We conducted the systematic literature search in the PubMed and Web of Science databases which include original full-text articles, randomized control trials and clinical trials, from 1 January 2012 up to 20 November 2022, published in English, with the “thrombophilia AND (pregnancy OR diagnostic criteria) AND fetal growth restriction” criteria. We identified 7 articles on PubMed and 65 articles on the Web of Science database. The exclusion criteria were duplicates, articles that lack originality, published in languages other than English, and on non-human populations. Two researchers, BMM and TS, extracted the included studies’ titles and abstracts, screened them for relevance for the present study theme and selected the relevant ones by performing cross-screening. As a result, 26 articles were selected, and after the bias assessment using the Newcastle–Ottawa Scale [15], 8 articles were included, as shown in Figure 1.

## 3. Results

Mirzaei et al. [16] included 25 women with singleton pregnancies diagnosed with intrauterine growth restriction (IUGR) and 25 women who delivered normal weight newborns in their case-control study. Researchers collected blood samples at 4 weeks postpartum and used genetic tests as well as biological tests. The thrombophilia prevalence was 32% in the control group and 68% in the IUGR group. A statistically significant association between inherited thrombophilia types and IUGR was present in the cases of MTHFR mutations, protein S deficiency and multiple thrombophilia (Table 1).

In a retrospective cohort study conducted by Berks et al. [17], 844 pregnant women with different phenotypes of preeclampsia were tested 6 weeks postpartum for inherited or acquired thrombophilia. They described a significant association between pregnant women with preeclampsia with more than one thrombophilia factor or antiphospholipid antibodies and IUGR (Table 1).

A case-control study published by Reshetnikov et al. [18] in 2017 included 497 pregnant women, among which 250 had FGR and 247 were controls. The study participants underwent DNA isolation followed by a genotyping assay. A significant association was found between polymorphism G>A FVII rs6046 and IUGR (Table 1).

In a prospective study, Mutlu et al. [19] screened 204 patients with previous poor obstetrical outcomes for thrombophilia mutations and coagulation panel, selecting the timing before their latest pregnancy. The only association between IUGR and inherited thrombophilia was with MTHFR mutation (Table 1).

A retrospective study published by Vicoveanu et al. [20] in 2021, which included 179 female pregnant patients with inherited thrombophilia, analyzed neonatal outcomes and concluded that FGR was statistically significantly associated with the homozygous mutation of factor V Leiden and combined MTHFR and factor V Leiden mutations (Table 1).

Kovac et al. [21] included 28 women who were genetically tested for specific mutations in the SERPING1 gene, which is responsible for different types of antithrombin deficiencies, while analyzing certain pregnancy and fetal outcomes in a retrospective cohort study. Homozygous type II HBS (heparin binding site), known as antithrombin Budapest 3, was linked to IUGR (Table 1).

Another retrospective case-control study published by Zemet et al. [22] enrolled 101 women with preeclampsia who delivered before or at 34 weeks of gestation. Of the 101 patients, the antiphospholipid status was only accessible for analysis for 55 women; 20 women had positive antiphospholipid antibodies. There was no statistically significant association between IUGR and the presence of antiphospholipid antibodies (Table 1).

A multicenter retrospective cohort study published by Saccone et al. in 2017 [23] performed in seven Italian University Hospitals included 750 pregnant women positive for antiphospholipid antibodies. Regarding the risk of IUGR, there is a 2.55-fold higher risk in pregnant women with positive antiphospholipid antibodies (Table 1).

The pathologies associated with thrombophilia that could play a role in developing IUGR, as mentioned in the articles included in our review, include preeclampsia [17,18,19,23], chronic hypertension [18,22], placental abruption [17,19,22], systemic lupus erythematosus [22], Behçet’s disease [22] and smoking [23].

## 4. Discussion

Thrombophilia represents a group of disorders that augment the risk of thromboembolic disease in the general population [24]. The risk is significantly increased in pregnant women due to the physiologic hypercoagulability caused by certain changes in coagulation factors. The diagnosis of inherited thrombophilia is realized using the following genetic tests: molecular analysis (factor V Leiden, prothrombin, MTHFR gene mutation) or biological non-molecular laboratory testing (antithrombin protein C, protein S deficiency) [25]. The relationship between thrombophilia and IUGR is still intensely debated in the literature. Identifying the thrombophilia type could represent an important step in properly diagnosing a pregnancy at risk of IUGR, as it could lead to active intervention in order to minimize or avoid the maternal–fetal complications of IUGR and reduce the risk of stillbirth [26].

Factor V Leiden mutation, prothrombin gene mutation and antithrombin deficiency are considered among the most representative thrombophilia disorders. The homozygote mutation of factor V Leiden, the heterozygote factor V Leiden mutation concomitant with prothrombin heterozygote gene mutation, homozygote prothrombin gene mutation and antithrombin deficiency are present in the high-risk classification of inherited thrombophilia [27]. Regarding the association with IUGR, there are studies which support a strong correlation between IUGR and different types of thrombophilia, while other studies support a low correlation; thus, in the current literature there is no consensus concerning the strongest association between the type of thrombophilia and IUGR. The discrepancies could be explained by the study population (for example factor V Leiden is the most frequent thrombophilia mutation in Europe; in the United States the prevalence is between 3% and 8%, while in Asia it is a rare mutation [28,29]) and also by the tested types of thrombophilia in each and every study. In our systematic review, one study [20], which tested patients for protein C activity, protein S activity, antithrombin III activity, factor VIII, active protein C resistance, prothrombin and active partial thromboplastin time, factor V Leiden, MTHFR C677T and A1298C as well as prothrombin G20210A mutations, revealed a significant association between factor V Leiden homozygote mutation, for the coexistence of factor V Leiden mutation with MTHFR mutation and IUGR, respectively; the prevalence of IUGR in these groups being between 33.3–83.4%. A meta-analysis realized by Dudding et al. [30] analyzed the correlation between factor V Leiden mutation and FGR in eight studies, yielding a 4.7 OR with 95% CI 2.3–9.5. Another meta-analysis published by Hemsworth et al. in 2016 [31], realized on 32 case-control and cohort studies, affirmed a 40% risk of FGR in pregnant women with factor V Leiden mutation. A meta-analysis by Howley et al. [32] conducted on 10 case-control studies found a significant association between factor V Leiden and IUGR with an OR of 2.7 with 95% CI 1.3–5.5. Concerning antithrombin deficiency, we included one study [21] which tested only the antithrombin mutation gene; the prevalence of IUGR in pregnant patients with antithrombin deficiency was 22%. A retrospective cohort study on 459 pregnant patients, performed by Voicu et al. [33], investigated the interrelationship between thrombophilia (protein C, antithrombin III, protein S, homocysteine, factor V Leiden, MTHFR, factor XIII and prothrombin G20210A gene mutations) and adverse pregnancy events, established a 60.37-fold risk of FGR in pregnant patients with antithrombin deficiency and an 11.69-fold risk of FGR in pregnant patients with prothrombin gene mutation compared to the control group (*p* < 0.05).

Two studies included in our research [16,19] have found a correlation between MTHFR mutations and IUGR, the reported prevalence of IUGR being between 48% and 90.9% in cases of pregnant women with MTHFR mutation. A systematic review and meta-analysis published by Bahrami et al. [34] in 2020 regarding the association between IUGR and MTHFR 677C>T polymorphism, which included eight studies with 687 cases of IUGR and 2336 controls, concluded that there is a significant association between MTHFR 677C>T polymorphism and IUGR with an OR 0.140, 95% CI 0.049–0.450 (*p* ≤ 0.001). In this direction, a case-control study by Dugalić et al. [35] containing 33 pregnant patients with IUGR of unknown cause tested for thrombophilia and 66 controls, and found a statistically significant link between IUGR and plasminogen activator inhibitor 1 (PAI-1) and MTHFR C677T homozygote mutation, with an OR 13.546 (CI 95% 3.79–48.37), *p* < 0.001, OR 8.139 (CI 95% 2.20–30.10), *p* = 0.002, respectively. On the other hand, there are studies that do not confirm the association of IUGR and MTHFR mutation. Del Gobbo et al. [36], in a study published in 2018 with 303 participants with MTHFR mutations and IUGR, further examined the placentas in order to determine the presence of altered DNA and did not find an association between MTHFR 1298CC genotype and IUGR, but found only a tendency to higher MTHFR 677TT in IUGR. Another review by Zhang et al. [37] which included 1896 cases and 3074 controls from 10 studies concerning the link between FGR and MTHFR C677T and A1298C mutations, determined that there is no relationship between FGR and MTHFR mutations with an OR 1.02, 95% CI 0.90–1.15.

One study included in our review [16] found a significant correlation between protein S deficiency and IUGR; there were more cases in the IUGR and protein S deficiency group compared to the control group (32% vs. 8% in the control group, OR 5.41% with 95% CI, *p* = 0.034). A systematic review published by Alfirevic et al. [38] in 2001 including 25 studies on thrombophilia and adverse pregnancy outcome, found three small studies, with a very small number of participants, debating the relationship between IUGR and protein S deficiency. Their analysis established a significant association of protein S deficiency in IUGR with an OR of 10.2 with 95% CI 1.1–91.0. In contrast, a retrospective cohort study by Fernández-Alba et al. [39] in 2017 containing 328 pregnant patients with IUGR and protein S deficiency treated with low molecular weight heparin and 11,884 patients in the control group, found no significant association between protein S deficiency and IUGR, the prevalence of IUGR in the protein S deficiency being 2.7%, while in the control group was 4.1%, the OR was 0.66, with 95% CI 0.34–1.28.

A study included in our review [18] describes the statistically significant association found in G>A FVII rs6046 polymorphism and IUGR; of the G, GG and GA alleles, the GG alleles having the strongest association with IUGR: OR 2.64 with 95% CI 1.71–4.09, *p* < 0.001. In the literature there is almost no data reported on this association.

In regard to acquired thrombophilia, of the relationship between antiphospholipid antibodies and IUGR, three studies were included in our final selection, their results being heterogenous: one retrospective cohort study [17] found a prevalence of antiphospholipid antibodies of 9.5% in the cases of IUGR, being statistically significant with a *p* < 0.01, while a retrospective case-control study [22] found no statistically significant association between the presence of antiphospholipid antibodies and IUGR. Finally, the results published by a multicenter retrospective cohort study [23], demonstrate the positive association with IUGR (40.8% cases of IUGR in patients with one positive antiphospholipid antibody and 53.6% in more than 1 positive antibody), with an OR of 2.55 with 95% CI 1.07–2.59, *p* < 0.01. Furthermore, the same study highlighted a correlation between antiphospholipid antibodies and severe preterm IUGR, as preterm severe IUGR was present in 13.4% of cases with one positive antiphospholipid antibody, in 28.2% of cases with more than 1 positive antibody with an OR of 2.09 with 95% CI 1.44–3.04, *p* < 0.01. A recent meta-analysis of 22 studies regarding the link between antibodies and the risk of fetal growth restriction published by Xu et al. [40] in 2022, has found a positive correlation between antiphospholipid antibodies and the risk of IUGR with an OR of 1.26 with 95% CI 1.12–1.40, anticardiolipin antibodies and IUGR with an OR of 2.25 with 95% CI 1.55–2.94 and anti-beta2 glycoprotein 1 antibodies and FGR with an OR of 1.31 with 95% CI 1.12–1.49. The same study did not obtain a statistically significant association between the presence of lupus anticoagulant and fetal growth restriction (OR 0.82 with 95% CI 0.54–1.10). ACOG maintains that the discrepancy in the literature regarding antiphospholipid syndrome and IUGR could be caused by the inclusion of pregnant women with low-positive antiphospholipid antibodies tests in studies [41].

Three studies included in our research investigated the connection between the presence of multiple thrombophilia and the risk of IUGR [16,17,20]. All three found a statistically significant correlation between multiple thrombophilia and the risk of IUGR, as the presence of IUGR in pregnant patients with more thrombophilia disorders was recorded in 24% to 83.4% of cases. A study published in 2004 by Tranquilli et al. [42] investigating adverse pregnancy outcomes in women with multiple thrombophilic factors has obtained a statistically significant association between the presence of multiple thrombophilia and IUGR compared to the presence of one thrombophilic factor (*p* < 0.001). In this direction, the diagnosis of thrombophilia is realized using genetic tests (factor V Leiden, MTHFR, factor VII, prothrombin, SERPING 1 gene mutations) or biological laboratory testing (protein C, protein S or antithrombin deficiency, antiphospholipid antibodies), having a clear role in predicting adverse pregnancy outcomes, especially fetal growth restriction, such as factor V Leiden mutation, MTHFR C667T mutation, protein S deficiency, antithrombin deficiency, factor VII polymorphism, antiphospholipid antibodies and the coexistence of multiple types of thrombophilia. Identifying the mutations, deficiencies or specific antibodies is an important step in managing pregnancies at risk, to determine the optimal treatment and to change the pregnancy surveillance from a low-risk pregnancy to a high-risk pregnancy.

When discussing treatment, there is a heterogenous opinion due to the lack of homogeneity regarding the inclusion criteria in studies and also in the main outcomes analyzed by investigators. For example, the secondary outcomes of the FRUIT-RCT [43,44], an international, multicenter randomized controlled trial, which included 139 pregnant women (<12 weeks of gestation) with an obstetrical history of previous delivery before 34 gestational weeks with hypertensive disorders and/or a small for gestational age (SGA) infant and inherited thrombophilia (protein S deficiency, protein C deficiency, factor V Leiden heterozygosity and prothrombin gene G20210A mutation heterozygosity, activated protein C resistance), either treated with 80 mg aspirin daily or low molecular weight heparin (LMWH), or dalteparin 5000 IU (weight adjusted dose) and aspirin 80 mg daily, concluded that although the use of LMWH and aspirin reduced the onset of hypertensive disorders before 34 gestational weeks, there was no difference in the incidence of fetal growth restriction between the two groups. In contrast, a study published by De Carolis et al. [45] on 38 thrombophilic women with histories of a poor obstetric outcome or thromboembolic events, who received 4000 IU enoxaparin daily, a dose established for patients with previous pregnancy complications (with the exception of one case who developed a skin reaction after using enoxaparin, further receiving nadroparin 0.3 mL daily), 6000 IU for patients with history of thromboembolic events, precisely the dose being doubled in two cases of patients with antithrombin deficiency and history of thromboembolic events, found a significant association between the birth weight increment in treated pregnancies compared with untreated pregnancies (*p* < 0.009). Even though the rate of IUGR was higher in the untreated group, at 27.7% vs. 7.9% in the treated group, statistical significance was not observed (*p* = 0.09); the explanation could the small number of participants in the study.

Women with an obstetrical history of FGR have a 20–30% risk of FGR recurrence in a future pregnancy [46]. Secondary prevention of FGR recurrence could be realized by counselling the patient into smoking cessation and recommending treatment with low-dose aspirin based on the first-trimester combined screening [47]. A prospective randomized control trial published by Orudzhova et al. [48] that included 32 patients with thrombophilia and a history of FGR treated with LMWH and low-dose aspirin concluded that managed treatment at the preconceptional and early pregnancy stages managed to prevent FGR recurrence in 78.1% of cases, while the presence of high antiphospholipid antibody titers could be a negative predictive factor for the prevention of FGR recurrence. At present, there are few available studies in the literature concerning the possible link between the presence of thrombophilia and FGR recurrence, a field that requires further research.

### Strengths and Limitations

The major strength of our work is represented by the fact that it is the most recent review of the literature concerning the association of the thrombophilic status and the risk of intrauterine growth restriction; our work also provides a comprehensive picture of the available data regarding the diagnosis of thrombophilia. We identified the following gaps in the scientific knowledge: there is no uniformity in the diagnosis of thrombophilia worldwide, there are very few data available concerning the recurrent risk of IUGR in cases of thrombophilia and there is a lack of a consensus regarding treatment in pregnancies complicated with thrombophilia, all of which could constitute subjects for future research. The primary limitation of our study is the fact that the literature search did not include all possible mutations implied in FGR (e.g., PAI-1 mutation, for which it is not clearly defined whether the connection between the thrombophilia diagnosis and fetal growth restriction is sustained). Another limitation may be the fact that the majority of the included studies, secondary to the protocol of this paper, are small studies with a limited number of patients; only one of the included works is a multicenter retrospective cohort study that enrolled 750 patients.

## 5. Conclusions

The diagnosis of thrombophilia is complex, including the biological elements and genetic elements that predict IUGR (factor V Leiden mutation, MTHFR C667T mutation, protein S deficiency, antithrombin deficiency, factor VII polymorphism, antiphospholipid antibodies), while other types of diagnosis are still under debate and require more data (protein C, PAI-1, certain combinations of mutations) and validation using larger randomized control trials. In addition, there is a need to classify thrombophilia according to the risk of venous thromboembolism as well as according to the fetal risks such as IUGR in order to improve the maternal–fetal outcome.

## Figures and Tables

**Table 1:** Summary of the results: thrombophilia diagnosis and type and fetal outcome, especially intrauterine growth restriction.

Author	Diagnosis	Thrombophilia Type	Fetal Outcome
Mirzaei et al. [16]	Genetic	MTHFR C667T mutation	IUGR 48% vs. normal 20%, OR 69% with 95% CI, *p* = 0.037
	Biological	Protein S deficiency	IUGR 32% vs. normal 8%, OR 5.41% with 95% CI, *p* = 0.034
	Genetic and biological	Multiple thrombophilia	IUGR 24% vs. normal 0, OR 2.1% with 95% CI 0–0.2, *p* = 0.009
Berks [17]	Genetic and biological	≥1 thrombophilia factor (antiphospholipid antibodies, APC-resistance, protein C deficiency and protein S deficiency, hyperhomocysteinemia, factor V Leiden and prothrombin gene mutation)	IUGR 36.8% vs. normal 25.1%, *p* < 0.01
	Biological	Antiphospholipid antibodies	IUGR 9.5% vs. normal 5.1%, *p* < 0.01
Reshetnikov [18]	Genetic	Polymorphism G>A FVII rs6046	G alleles: IUGR OR 2.34 with 95% CI 1.60–3.44, *p* < 0.001 GG alleles: IUGR OR 2.64 with 95% CI 1.71–4.09, *p* < 0.001 GA alleles: IUGR OR 0.42 with 95% CI 0.27–0.64, *p* < 0.001
Mutlu [19]	Genetic	MTHFR mutations (C677T and A1298C)	IUGR in 90.9% cases
Vicoveanu [20]	Genetic	Homozygous mutation of factor V Leiden	IUGR in 33.3% cases, *p* = 0.04
		Factor V Leiden and MTHFR mutations	IUGR in 83.4% cases, *p* = 0.02
Kovac [21]	Genetic	Homozygous type II HBS (Antithrombin Budapest 3)	IUGR in 22% cases
Zemet [22]	Biological	Antiphospholipid antibodies (anticardiolipin antibodies, β2 Glycoprotein1 antibodies or lupus anticoagulant)	IUGR in 25% cases vs. normal 17.1%, *p* = 0.7
Saccone [23]	Biological	Antiphospholipid antibodies (anticardiolipin antibodies, β2 glycoprotein1 antibodies or lupus anticoagulant)	IUGR in 40.8% cases with single positive antiphospholipid antibody and 53.6% in more than 1 positive antibody—OR 2.55 with 95% CI 1.07–2.59, *p* < 0.01 Severe preterm IUGR in 13.4% cases with single positive antiphospholipid antibody and 28.2% in more than 1 positive antibody—OR 2.09 with 95% CI 1.44–3.04, *p* < 0.01

MTHFR—methylenetetrahydrofolate reductase; IUGR—intrauterine growth restriction; OR—odds ratio; CI—confidence interval; HBS—heparin binding site; APC—activated protein C; FVII—coagulation factor VII or proconvertin.

## Data Availability

Not applicable.
